# Sestrin2 in hypoxia and hypoxia-related diseases

**DOI:** 10.1080/13510002.2021.1948774

**Published:** 2021-07-05

**Authors:** Xiaojing Che, Jiagui Chai, Yan Fang, Xifeng Zhang, Anju Zu, Lin Li, Shibo Sun, Weimin Yang

**Affiliations:** a Department of Pulmonary and Critical Care Medicine, First Affiliated Hospital, Kunming Medical University, Kunming, People’s Republic of China; b Innovation Class & Second Class, 2017 Clinical Medicine, Kunming Medical University, Kunming, People’s Republic of China; c School of Pharmaceutical Science & Yunnan Key Laboratory of Pharmacology for Natural Products, Kunming Medical University, Kunming, People’s Republic of China

**Keywords:** Sestrin2, hypoxia, reactive oxygen species (ROS), ischemia/reperfution (I/R), cardiomyocyte, hypoxia-related diseases, chronic obstructive pulmonary disease (COPD), obstructive sleep apnea (OSA)

## Abstract

**Objectives:** Sestrin2 is a stress-inducible protein and play an important role in adapting stress states of cells. This article reviewed the role of Sestrin2 in hypoxia and hypoxia-related diseases to provide new perspectives for future research and new therapeutic targets for hypoxia-related diseases.

**Methods:** A review was conducted through an electronic search of PubMed and Medline databases. Keywords included Sestrin2, ROS, hypoxia, and hypoxia-related disease. Articles from 2008 to 2021 were mostly included and older ones were not excluded.

**Results:** Sestrin2 is upregulated under various stress conditions, especially hypoxia. Under hypoxic condition, Sestrin2 plays a protective role by reducing the generation of ROS through various pathways, such as adenosine monophosphatea-ctivated protein kinase (AMPK) / mammalian target of rapamycin (mTOR) pathway and nuclear factor-E2-related factor2 (Nrf2) pathway. In addition, Sestrin2 is involved in various hypoxia-related diseases, such as cerebral hypoxic disease, myocardial hypoxic disease, hypoxia-related respiratory disease, and diabetes.

**Discussion:** Sestrin2 is involved in various hypoxia-related diseases and maybe a therapeutic target. Furthermore, most studies focus on cerebral and myocardial ischemia reperfusion. More researches on hypoxia-related respiratory diseases, kidney injury, and diabetes are needed in future.

## 1. Introduction

Sestrins are a family of highly conserved stress-inducible proteins and play important roles in adapting stress states of cells [1,2]. In mammals, Sestrins include three subtypes: Sestrin1, Sestrin2, and Sestrin3 [3,4]. Sestrin1, also known as p53-activated gene 26 (PA26), is involved in cell growth and regulating tumor, which is induced by serum starvation and growth arrest [3]. Sestrin2, a homolog of PA26, has been considered to be related to hypoxia, endoplasmic reticulum (ER) stress, oxidative stress, starvation, and DNA damage [4,5]. Sestrin3, a PA26 structure-related gene, is confirmed to be related to energy crisis, which is induced by the forkhead box O (FoxO) to maintain the cellular energy stores during oxidative challenge [1].

**Figure 1.**
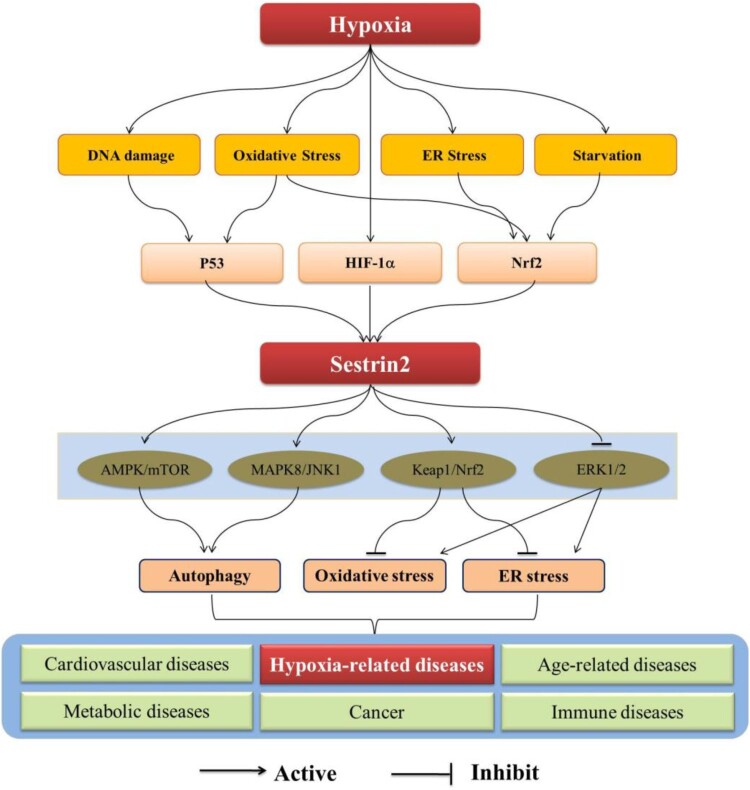
Signaling pathways associated with Sestrin2. Note: ER, endoplasmic reticulum; HIF-1α, hypoxia inducible factor-1α; Nrf2, nuclear factor-E2 -related factor 2; AMPK, adenosine monophosphate-activated protein kinase; mTOR, mammalian target of rapamycin; MAPK8/JNK1, mitogen-activated protein kinases 8/c-Jun N-terminal kinase 1; Keap1, kelch-like ECH-related protein 1.

**Figure 2.**
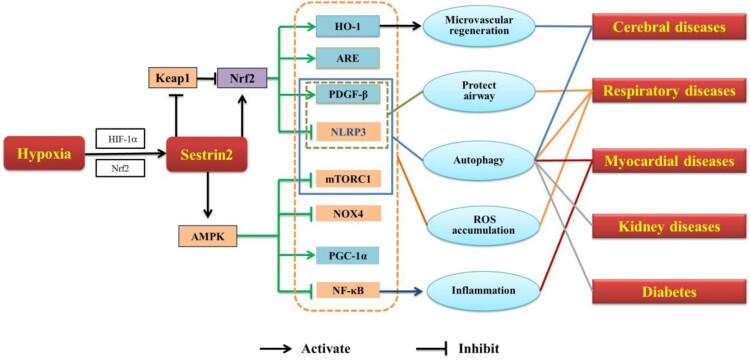
Sestrin2 in hypoxia-related diseases. Note: HIF-1α, hypoxia inducible factor-1α; Nrf2, nuclear factor-E2-related factor 2; Keap1, kelch-like ECH-related protein 1; AMPK, adenosine monophosphate-activated protein kinase; HO-1, heme oxygenase-1; ARE, antioxidant reaction element; PDGF-β, platelet-derived growth factor receptor β; NLRP3, nod-like receptor protein 3; mTORC1, mammalian target of rapamycin complex 1; NOX4, nicotinamide adenine dinucleotide phosphate oxidase; PGC-1α, proliferator-activated receptor γ coactivator-1α; NF-κB, nuclear factor-kappaB; ROS, reactive oxygen species.

Currently, increasing studies focus on the relationship between hypoxia and Sestrin2 now that the hypoxia is an important inducer of stress [6,7]. Diverse pathways are subsequently confirmed to be involved in this relationship (Figure 1). Meanwhile, Sestrin2 is demonstrated to be related with several hypoxia-related diseases, such as cerebral disease, myocardial disease, respiratory diseases, and so on [8–11] (Figure 2). Increasing evidences suggest that Sestrin2 may be a promising therapeutic target for the treatment of these hypoxia-related diseases [12–15]. Accordingly, we summarized the current research progresses regulatory mechanisms of Sestrin2 under hypoxia and its potential therapeutic targets in hypoxia-related diseases.

## 2. Pathways associated with Sestrin2 under hypoxia

Recently, studies suggest that the Sestrin2 is regulated by several factors, such as P53, hypoxia inducible factor-1α (HIF-1α) and nuclear factor-erythroid 2-related factor2 (Nrf2), to exert a cells protective effect through promoting autophagy, inhibiting oxidative stress and ER stress [16–19]. In this protective process, several pathways are activated, such as adenosine monophosphate-activated protein kinase/mammalian target of rapamycin (AMPK/mTOR) pathway [20,21], nuclear factor-E2-related factor2/Kelch-like ECH-related protein1 (Nrf2/Keap1) pathway [22,23], the mitogen-activated protein kinases8/c-Jun N-terminal kinase1 (MAPK8/JNK1) pathway [19,24], AMPK / PGC-1α pathway [8], and ERK1/2 pathway [25]. Among of those pathways, the AMPK/mTOR and Nrf2/Keap1 are the key pathways that the Sestrin2 is involved under hypoxia.

It has been widely reported that hypoxia-related diseases are related to Sestrin2 [12,26]. In hypoxia-related diseases, hypoxia leads to imbalance of redox homeostasis because of excessive accumulation of reactive oxygen species (ROS) [27], which causes dysfunction of cells [14]. In addition, hypoxia activates HIF-1α, resulting in the activation of Sestrin2 [28–30]. Sestrin2 downregulates the mTOR directly or through AMPK to promote autophagy or reduce ROS generation to maintain cell redox balance [21,31]. Similarly, the activation of AMPK induced by Sestrin2 also inhibits the nicotinamide adenine dinucleotide phosphate oxidase 4 (NOX4) and the regeneration of NOX4-dependent ROS [32]. Accordingly, AMPK/mTOR is a key signaling pathway of the Sestrin2.

Furthermore, Sestrin2 promotes the activation of Nrf2 and degradation of Keap1 to exert antioxidant roles [22,23]. It was reported that overexpression of Sestrin2 may promote angiogenesis by activating the Nrf2 pathway through increasing the interaction between p62 and Keap1 to improve the neurological function and reduce the infarct volume and brain edema of ischemic stroke rats [33]. In addition, downregulating Sestrin2 results to activation of Nrf2 and upregulation of Keap1, which causes the myocardial remodeling. Meanwhile, rectifying the abnormal expression of Sestrin2/Nrf2/keap1 significantly ameliorates cardiac remodeling [9]. Accordingly, Nrf2/keap1 is another important pathway of Sestrin2.

## 3. Sestrin2 and hypoxia-related diseases

### 3.1. Sestrin2 and cerebral hypoxic disease

The neuronal cells are specifically assailable to hypoxia because of the unique structure and function, such as abundant presence of polyunsaturated fatty acid, high metabolism and dependence on oxygen [34]. Consequently, neuronal DNA damage or protein destruction is easily caused by hypoxia due to the accumulation of ROS [35]. Therefore, reducing excessive ROS is crucial to protect neuronal cells from the injury under hypoxia. It is reported that Sestrin2, as an antioxidant, exerts neuroprotective effects through various pathways under hypoxia [12,36–38]. Accordingly, Sestrin2 is closely related to the cerebral hypoxic disease.

Increasing studies suggest that Sestrin2 has important antioxidant effects in cerebral ischemia/reperfution (I/R) injury. The activation of Sestrin2 not only activates the AMPK pathway to inhibit mTORC1 and NOX4 to decrease the ROS accumulation [12,32,39], but also activates the monophosphate-activated protein kinase/peroxisome proliferator-activated receptor γ coactivator-1α (AMPK/PGC-1α) pathway to promote mitochondrial biogenesis and improve mitochondrial biological activity, thereby reducing the generation of ROS [8]. In addition, Sestrin2 which is upregulated by brain-derived neurotrophic factor (BDNF) exerts neuronal protection by the pathway of nitric oxide/3’, 5'-cyclic guanosine monophosphate-dependent protein kinase/nuclear factor-kappaB (NO/PKG/NF-κB) [40]. Moreover, Sestrin2 promotes the degradation of Nrf2 inhibitor by up-regulating the expression of scaffold protein p62, to limit neuropathic pain processing by antioxidant effect [33,41]. Meanwhile, Sestrin2 upregulates downstream factors of Nrf2, such as sulfiredoxin1 (Srx1) and thioredoxin1 (Trx1), to resist neuronal injury [17]. Besides that, Sestrin2 is induced by miR-148b-3 inhibition to enhance the activation of Nrf2/antioxidant reaction element (ARE) antioxidant signaling to inhibit oxidative stress [42,43]. Furthermore, Sestrin2 appears to protect neuronal cells against I/R by down-regulating the phosphorylation of ribosomal protein S6 kinase (SK6) via negative regulation of mTORC1 [44].

In addition, many studies illustrate that Sestrin2 protect neuronal cells by promoting angiogenesis. Wang et al. [38] have demonstrated that Sestrin2 upregulated the vascular endothelial growth factor (VEGF) to promote microvascular regeneration and reduce cerebral ischemic damage in the cerebral ischemic area through Nrf2/heme oxygenase-1 (HO-1) pathway. However, Shi et al. [45] found that the severity of cerebral ischemia acted differently on Sestrin2 expression levels. Sestrin2 is significantly induced by HIF-1α in severe cerebral ischemia and inhibits VEGF to reduce the permeability of blood–brain barrier (BBB), which reduces the occurrence of cerebral edema and hemorrhagic cerebral infarction [45–47]. Taken together, Sestrin2 plays a significant role in cerebral hypoxic disease, it may serve as a potential therapeutic target for brain I/R injury.

### 3.2. Sestrin2 and myocardial hypoxic disease

Myocardial hypoxia is considered to be a characteristic of the failing heart [47]. Continuous myocardial hypoxia increases the production of ROS, leading to DNA damage and Ca^2+^ overload of cardiomyocytes or mitochondria, resulting in cell death [48,49]. Additionally, hypoxia also inhibits mitochondrial oxidative phosphorylation of cardiomyocytes, leading to insufficient adenosine triphosphate (ATP) production, which causes intracellular acidosis and weakens myocardial contractility. The ROS accumulation and ATP lack are important factors of cardiomyocyte damage. Accordingly, many researchers have explored the antioxidant role of Sestrin2 in the process of myocardial hypoxia [50–52].

Present studies suggest that Sestrin2 plays an important role in cardioprotection against I/R injury, serving as an LKB1-AMPK scaffold to initiate AMPK activation during ischemic insults [53]. Sestrin2 recognizes damaged mitochondria and improves the autophagy efficiency of cardiomyocytes to reduce the generation of ROS by activating AMPK to inhibit mTORC1 [12,54,55]. In addition, Sestrin2 activates AMPK to stimulate cardiomyocyte mitochondrial biogenesis by regulating PGC-1α [50]. Furthermore, the accumulation of ROS induces the imbalance of nitric oxide and nitric oxide synthase (NOS-NO), resulting in the damage of cardiomyocytes [56]. The upregulation of Sestrin2 is likely to activate guanylate cyclase through the NOS-NO-cyclic guanosine monophosphate (cGMP) signaling pathway to protect the heart by causing vasoconstriction and reducing the load of the heart and infarct size [57].

Moreover, hypoxia causes the filtration of inflammatory factors which leads to dysfunction of the heart because of aseptic inflammation of cardiomyocytes [58]. It is reported that Sestrin2 appears to inhibit cell membrane inflammation and lipid peroxidation chain reaction by downregulating NF-κB activation, JNK signal and interleukin-1β (IL-1β) expression, so as to reduce myocardial tissue damage [51]. Overall, Sestrin2 may provide a new idea for therapeutic strategies to treat myocardial hypoxic disease.

### 3.3. Sestrin2 and hypoxia-related respiratory diseases

Some respiratory diseases are usually accompanied by airway inflammation and airway obstruction, which lead to physiological dysfunction of the lung, and then make the body in a state of hypoxia [59,60]. Therefore, hypoxia is considered to play an important role in the process of hypoxia-related respiratory diseases.

At present, increasing studies have found that Sestrin2 may exert a protective effect on hypoxia-related respiratory diseases. On the one hand, Sestrin2 maintains the integrity of airway epithelial cells [6]. On the other hand, Sestrin2 protects cells from oxidative damage [61]. Thus authors suggested that Sestrin2 participates in the pathological process of respiratory diseases [6,61].

The role of Sestrin2 in COPD is highly complex. The inactivation of Sestrin2 increases the expression of PDGFR-β by inhibiting Nrf2/Keap1 pathway in a TGF-β-dependent manner to improve COPD by inhibiting oxidative stress or maintaining the integrity of airway epithelial cells [10,62,63]. Surprisingly, the activation of Sestrin2 also protects the integrity of airway epithelial barrier [6]. Both activation and inactivation of Sestrin2 play a protective role in COPD. We speculate that the double protective roles of Sestrin2 may be related to HIF-1α, because HIF-1α, as a regulator of the expression of Sestrin2, has different effects on different degree of hypoxia [15,64].

In addition, the level of Sestrin2 is significantly higher in plasma and urine of OSA patients, and is positively correlated with AHI, even is helpful to the diagnosis of OSA [27,65,66]. Moreover, the level of Sestrin2 is lower after treatment of CPAP which can effectively alleviate intermittent hypoxia and oxidative stress in OSA patients [66]. This suggests that the higher level of Sestrin2 in OSA patients may be caused by intermittent hypoxia and oxidative stress, which may be related to antioxidant effects of Sestrin2. However, the specific mechanism is still unclear.

It is reported that the level of Sestrin2 is significantly higher in asthma patientsand is independently correlated with the FEV1% predicted [67]. It suggests that Sestrin2 may be a marker of uncontrolled asthma. However, the relationship between Sestrin2 and asthma needs to be further explored.

### 3.4. Sestrin2 and other hypoxia-related diseases

Acute kidney injury (AKI) is a clinical syndrome with high incidence rate and poor prognosis [68,69]. It is suggested that AKI is mainly caused by renal cell apoptosis because of renal I/R [70]. It is reported that Sestrin2 is overexpressed in animal or cell model of AKI and the overexpression is beneficial to AKI [54].

Accumulating evidence suggests that hypoxia is an important pathogenic factor for diabetes or insulin resistance [71–73]. Sestrin2 is important to maintain insulin sensitivity and glucose metabolism through HIF-1α or AMPK-dependent autophagic activation [72,74]. It was reported that circulatory Sestrin2 was decreased in diabetes and negatively correlated with glycemic levels [75,76]. However, these decreases were not found in the study from Chung et al. [77]. Accordingly, the level of circulatory Sestrin2 is still controversial.

## 4. Conclusion

Sestrin2, as highly conserved stress-inducible protein, is involved in various hypoxia-related diseases and plays a protective role mainly through reducing production of ROS. The related pathways mainly include AMPK-mTOR, Keap1-Nrf2, Nrf2-HO-1 and other pathways. Therefore, Sestrin2 appears to be a therapeutic target for hypoxic-related diseases. Currently, most studies focus on cerebral and myocardial ischemia reperfusion. More researches on hypoxia-related respiratory diseases, kidney injury and diabetes are needed in future.
